# Identification of human milk oligosaccharide positional isomers by combining IMS-CID-IMS and cryogenic IR spectroscopy

**DOI:** 10.1039/d3an00407d

**Published:** 2023-04-21

**Authors:** Ali H. Abikhodr, Ahmed Ben Faleh, Stephan Warnke, Vasyl Yatsyna, Thomas R. Rizzo

**Affiliations:** a Laboratoire de Chimie Physique Moléculaire, École Polytechnique Fédérale de Lausanne, EPFL SB ISIC LCPM Station 6 CH-1015 Lausanne Switzerland thomas.rizzo@epfl.ch

## Abstract

High-resolution ion mobility spectrometry (IMS) coupled with cryogenic infrared spectroscopy has proven to be a powerful technique for the identification of oligosaccharides. However, the need for an extensive database, combined with the scarcity of pure standards, remains a significant barrier to the broad application of this approach. To solve this issue, we demonstrate a method in which ion fragments produced by collision-induced dissociation (CID) are separated using IMS and identified using the vibrational fingerprints of only a few standards. Identification of the fragments allows us to determine the structure of the precursor molecule, the vibrational fingerprint of which is then added to our database. We then show how we can use this approach to identify the structure of mobility separated isomers found in pooled human milk.

## Abstract 2

Cryogenic infrared spectra of CID fragments separated by IMS are used to identify human milk oligiosaccharide isomers.

## Graphical abstract

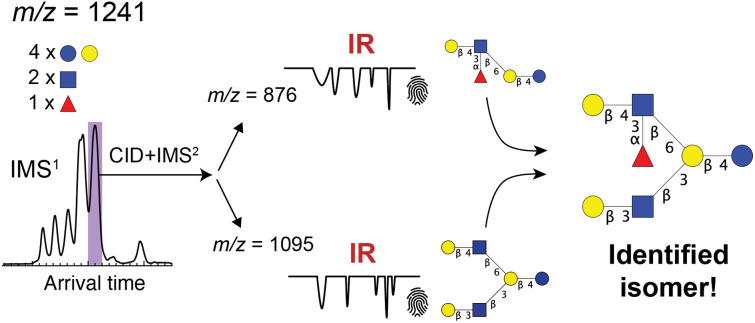

## Introduction

Human milk oligosaccharides (HMOs) play an essential role in developing the immune system of infants by stimulating healthy gut microbiota.^1–4^ Although they are composed of combinations of only five monosaccharides (glucose, galactose, *N*-acetylglucosamine, fucose, and sialic acid), they exhibit an extreme degree of structural complexity. The majority of HMOs consist of a lactose core (Gal-β1,4-Glc) at the reducing end, which is then extended *via* a β1-3 bond with either lacto-*N*-biose I (Gal-β1,3GlcNAc) or lactosamine (Gal-β1,4-GlcNAc). Branching can occur through β1-6 bonding between GlcNAc and the Gal of lactose. Further modifications arise from an alpha 1-2,3,4 bonded fucose or alpha 2-3,6 bonded sialic acid.^5^ This intrinsic isomeric complexity poses a formidable challenge for the analysis of HMO primary structure.^6,7^

Various analytical techniques have been employed for oligosaccharide structural analysis. Tandem mass spectrometry (MS) uses fragmentation patterns to reveal molecular structure,^8–10^ however, this approach has difficulties distinguishing subtly different isomers. The use of orthogonal separation techniques in combination with tandem MS, such as liquid chromatography (LC) and ion mobility spectrometry (IMS), can help solve this problem. Both LC methods^11–13^ and high-resolution IMS approaches,^14–16^ have demonstrated the ability to separate many oligosaccharide isomers, however assigning their precise isomeric form remains challenging.^17,18^

In our laboratory, we couple high-resolution IMS with cryogenic infrared (IR) spectroscopy^19–21^ to measure IR fingerprints of separated isomers and use these spectra as identifiers *via* a database approach. The advantages of using an IR fingerprint for identification are twofold: (1) it is extremely sensitive to the slightest difference in structure and hence unique to a given isomer; and (2) it is an intrinsic property of the molecule, which makes it a robust metric.^22^ One drawback of this approach is its reliance on the existence of an extensive IR database of analytical standards, which are often unavailable and difficult to synthesize.

To tackle this issue, we have developed a procedure based on tandem IMS in combination with cryogenic IR fingerprinting^23^ to identify glycans for which we do not have standards.^24^ In brief, isomeric fragments generated by CID are separated by IMS and identified by their IR fingerprints, using a limited number of standards. Knowledge of the structure of these fragments is then used to reconstruct the primary structure of the precursor molecule. Once the precise isomeric form of the precursor is identified, its IR spectrum can be used to expand the IR database. This approach is especially well suited for human milk oligosaccharides due to the nature of their biosynthesis in which larger species are built off a lactose core by enzymes with high regio- and stereoselectivity.^25–27^

To illustrate the feasibility of this approach, we first apply it to a fucosylated human milk oligosaccharide, where we identify the position and linkage of the fucose residues. While many of these oligosaccharides can be identified by LC-MS/MS, which combines information from the retention time and MS/MS fragmentation data,^28,29^ their identification is column- and technique-specific, which means that the identification process must be repeated if the LC/fragmentation method is changed. In contrast, because a vibrational spectrum is an intrinsic property of the analyte molecule, the identification procedure described above has only to be performed once to acquire the parent IR fingerprint, after which IR spectroscopy alone is sufficient for identification. We then use this method to identify oligosaccharides extracted from a pooled human milk sample for which we had no entries in our infrared spectral database. We demonstrate how we can assign peaks in a complex arrival-time distribution (ATD) to specific isomers.

## Experimental methods

### Sample preparation

The human milk oligosaccharide standards employed in this work (LNFP I, LNFP II, LNH, LNDFH I, MFLNH I, and DFLNH b) were acquired from Carbosynth Ltd and Dextra Laboratories Ltd. All standard solutions were prepared in a 50/50 mixture of water/methanol and used without further purification at a concentration of 10–25 μM. All samples were analyzed in the sodiated charge state to avoid fucose migration, which occurs in the protonated state.^30^

A pooled human milk sample (0.5 mL lyophilized), purchased from Chemie Brunschwig AG, was reconstituted in 0.5 mL of water. We then centrifuged 50 μL of this sample at 13 000 rpm for 30 min at 4 °C to de-fat the solution. The resulting aqueous fraction was mixed with ethanol (1 : 1) and held at −80 °C for 1 h, followed by 30 minutes of centrifugation at 13 000 rpm and 4 °C to precipitate the proteins. The supernatant containing HMOs was then diluted 60 times before analysis on our instrument.

### Instrumentation

All experiments in this work were performed using a home-built instrument described in detail elsewhere.^31^ It includes high-resolution ion mobility separation using structures for lossless ion manipulations (SLIM)^16,32^ and a cryogenic trap coupled to a time-of-flight (TOF) mass spectrometer (TOFWERK) for IR spectroscopy. Briefly, ions are produced by nano-electrospray (nESI) and introduced into the instrument through a heated stainless-steel capillary (130° C). Initially, the ions are guided through a set of ion funnels and accumulated in an 2m long accumulation segment on the SLIM device operating with traveling-wave frequency of 9900 Hz with an amplitude of 14 V_p–p_. Ion packets of 1 ms duration are then released and guided through the SLIM-IMS separation region, which is held at a nitrogen pressure of 2.2 mbar. The SLIM device we designed has a single-pass path length of 10 m and can be operated in cyclic mode to increase the resolution. It also includes a trapping section in which we perform CID on mobility selected ions.^24^ While the conditions for optimal separation depend on the *m*/*z* we are analyzing, the traveling-wave frequency is on the order of 19 800 Hz with and amplitude of 42 V_p–p_.

Following separation, mobility-selected ions are guided through multiple differential pumping stages to the cryogenic trap, which is maintained at 45 K in order to perform messenger-tagging IR spectroscopy. Trapped ions are cooled *via* collisions with a He/N_2_ (80 : 20) gas mixture and form weakly bound clusters with N_2_ molecules. The N_2_-tagged ions are irradiated with a continuous-wave mid-IR laser (IPG Photonics) for 50 ms and analyzed using the TOF mass spectrometer. Upon absorption of an IR photon, energy is redistributed among the vibrational modes, leading to dissociation of the nitrogen tag(s). We obtain an IR fingerprint spectrum of the tagged species by measuring the ratio between the number of tagged and untagged ions at each laser wavenumber step. While we can measure a spectrum in as little as 7 seconds, in this work we typically average two individual 7-minute scans to improve the S/N.

### HMO IR fingerprint database

Infrared spectra of LNFP I and II have been previously measured and added to our database.^33^ For the work reported here, we measured IR spectra of three additional standards, LNH, DFLNH b (and its fragments), and MFLNH I. This database allows us to directly detect the presence of these species in complex mixtures as intact molecules or as fragments of larger unknown molecules.

### Spectral comparison

In this study, we use the Pearson correlation coefficient (PCC) to compare measured infrared (IR) spectra with those in our database.^34–36^ The PCC method measures the linear relationship between two vector variables, and then provides a value ranging from −1 to 1. A coefficient close to 1 indicates a strong positive correlation, while a value close to −1 signifies a strong negative correlation, and a value around 0 implies no correlation. By calculating the PCC for our measured IR spectrum with each of our reference spectra, we can determine the degree of similarity between them and hence the best match. In this work, all identified spectra had a PCC ranging between 0.92 and 0.97, which indicates very good agreement.

## Results and discussion

### IR identification of CID fragments

**Fig. 1:**
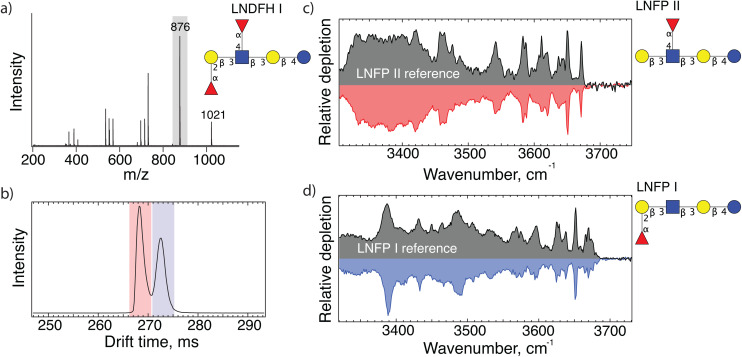
(a) MS/MS spectrum of sodiated lacto-*N*-difucohexaose I (LNDFH I structure shown); (b) arrival time distribution of 876 *m*/*z* fragments after 10 m SLIM-IMS separation; (c and d) messenger- tagging IR spectra corresponding to individual arrival time peaks shaded red and blue in panel (b) compared with database spectra.

As a test to demonstrate the principle of our approach for identifying human milk oligosaccharides, we first investigate lacto-*N*-difucohexaose I (LNDFH I, *m*/*z* 1021). Fig. 1(a) shows the CID fragment mass spectrum of an LNDFH I standard. The major fragment (*m*/*z* 876) corresponds to the loss of a single fucose, leaving three hexoses, one GlcNAc, and one fucose behind. The ATD of this fragment shows two major peaks (Fig. 1(b)), each of which we direct into our cryogenic ion trap and measure their respective IR spectrum, shown in Fig. 1(c and d). The good agreement between the spectrum of each mobility peak with those found in our database allows us to identify the two isomeric fragments as LNFP II and LNFP I.

The presence of the LNFP II fragment reveals the position of a fucose on the *N*-acetyl glucosamine (GlcNAc) *via* an (α1-4) bond, whereas the LNFP I fragment identifies a fucose bound to the terminal galactose (Gal) *via* an (α1-2) bond. Moreover, both fragments share the same backbone. Combining this information allows us to correctly deduce the structure of the precursor molecule, LNDFH 1.

### Characterization of a selection of oligosaccharides extracted from human milk

**Fig. 2:**
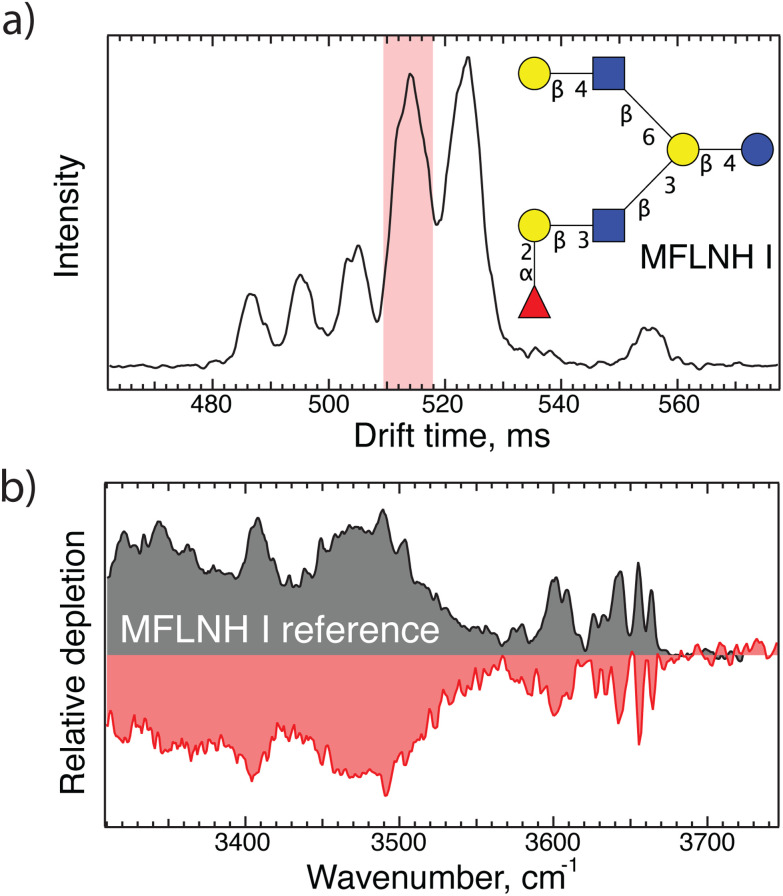
(a) Arrival time distribution of an HMO with *m*/*z* 1241 from human milk after 30 m SLIM-IMS separation; (b) IR fingerprint of the highlighted peak in panel (a) shown in red, and the corresponding database spectrum of MFLNH I shown in grey.

Having demonstrated the principle of our fragment-based identification approach on an LNDFH I standard where we already knew the structure, we now show its applicability to a complex pooled human milk sample from which we extracted the glycan content as described above. The arrival time distribution of glycans of *m*/*z* 1241 after three separation cycles (30 m drift length), shown in Fig. 2(a), consists of at least six different features, emphasizing the isomeric complexity of HMOs. As shown in Fig. 2(b), we can directly identify the isomer responsible for the peak arriving at 514 ms *via* spectral comparison with our database to be MFLNH 1, the structure of which is shown as an inset in Fig. 2(a).

**Fig. 3:**
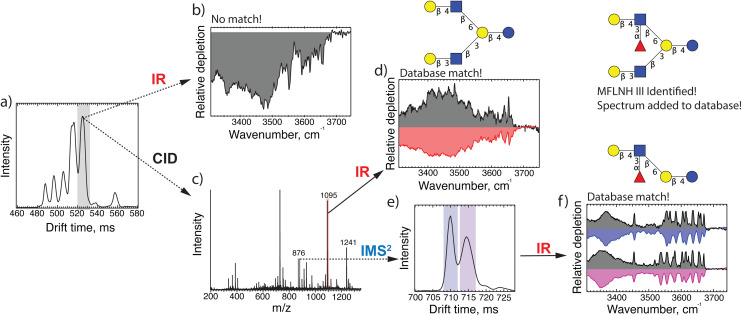
(a)ATD of *m*/*z* 1241 from human milk; (b) IR fingerprint of the highlighted peak in panel (a); (c) fragment mass spectrum of the highlighted peak in panel (a); (d) IR fingerprint of *m*/*z* 1095; (e) ATD of *m*/*z* 876 fragment after 10 m drift path; (f) IR fingerprint of both peaks in panel (e) (in color) compared with spectra from our IR database (in grey).

While this identification was straightforward, the IR fingerprint of the isomer arriving at 525 ms (shown in Fig. 3(b)) did not match any entry in our database. We therefore selectively performed CID to generate diagnostic fragments, the mass spectrum of which is displayed in Fig. 3(c). Using our IR database, we can assign the *m*/*z* 1095 fragment, which corresponds to the loss of one fucose, to the oligosaccharide LNH, shown in Fig. 3(d). Furthermore, upon mobility separation of the *m*/*z* 876 fragment, which is composed of three hexoses, one GlcNAc and one fucose, we observe two isomeric structures, as shown in Fig. 3(e). Based on matches of their IR spectra with entries in our database, shown in Fig. 3(f), we identify these isomers as the two reducing-end anomers of the glycan Gal(β1-4)[(Fuc(α1-3)]GlcNAc(β1-6)Gal(β1-4)Glc. Putting all this information together, we can assign the structure of the precursor (*m*/*z* 1241) as having an LNH backbone with a fucose α1-3 linked to the top branch GlcNac, which is MFLNH III. We thus add its IR fingerprint (shown in Fig. 3(b)), which we assigned without the need for a pure analytical standard for this isomer, to our database.

**Fig. 4:**
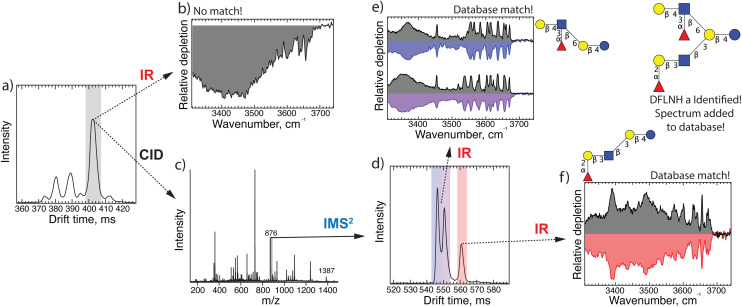
(a) Arrival time distribution of the HMO with *m*/*z* 1387 extracted from human milk after 20 m SLIM-IMS separation; (b) IR fingerprint of the highlighted peak in panel (a); (c) fragment mass spectrum of the highlighted peak in panel (a); (d) ATD of *m*/*z* 876 fragment after 10 m IMS separation; (e) IR fingerprint of the first two peaks in panel (d) (in color) and their comparison with database spectra (in grey); (f) IR fingerprint of the third peak in panel (e) (red) and its comparison with database spectrum (grey).

Fig. 4(a) displays the ATD of another HMO (*m*/*z* 1387) from the pooled milk sample, which after two cycles (20 m) of separation appears to consist of three major isomers. The IR spectrum of the intense peak arriving at 403 ms (Fig. 4(b)) did not match those already in our database, and thus required application of our fragmentation scheme. Fig. 4(c) shows the fragment mass spectrum of the mobility selected peak, and Fig. 4(d) displays the ATD (one separation cycle) of the *m*/*z* 876 fragment, where we observe three features. Using cryogenic IR spectroscopy, we can assign the first two peaks in the ATD to the reducing-end anomers of the oligosaccharide Gal(β1-4)[(Fuc(α1-3)]GlcNAc(β1-6)Gal(β1-4)Glc and the third peak as LNFP I (*i.e.*, Fuc(α1-2)Gal(β1-3)GlcNAc(β1-3)Gal(β1-4)Glc), the structures of which are shown in Fig. 4(e and f), respectively. Both of these feature a common lactose core (Gal(β1-4)Glc). The one shown in Fig. 4(f) has Fuc(α1-2)Gal(β1-3)GlcNAc connected to the non-reducing end of lactose *via* a β1-3 bond. This intact oligosaccharide is not found in human milk, but we had generated it as a fragment from a larger HMO and measured its IR spectrum. The structure in Fig. 4(e) indicates a Gal(β1-4)[(Fuc(α1-3)]GlcNAc branched from lactose *via* a β1-6 bond. Putting these together, we identify the precursor structure to be (Fuc(α1-2)Gal(β1-3)GlcNAc(β1-3)[Gal(β1-4)[(Fuc(α1-3)]GlcNAc(β1-6)]Gal(β1-4)Glc), which is called DFLNH a and shown in the upper right hand corner of Fig. 4. We thus add its IR fingerprint (Fig. 4(b)) to our database without having had the need of a pure analytical standard for this species.

## Conclusion

We have combined IMS-CID-IMS with cryogenic IR spectroscopy to identify peaks in the arrival-time distributions of HMO isomers without having had analytical standards for the precursor molecules. We first applied our approach to LNDFH I to verify that we can determine the correct location of fucose residues. We then assigned features in the ATDs of HMOs from a pooled human milk sample with *m*/*z* 1241 and 1387 as corresponding to the isomers MFLNH III and DFLNH a, respectively, and expanded our IR database with their spectra. Future encounter of these molecules can then be identified by IR spectroscopy alone. The database is also expanded by fragmenting these oligosaccharide structures and acquiring the IR fingerprints of the fragments.

This approach has the potential to have a major impact in applications that require routine identification of molecules, such as in drug development and biomarker research.

## Conflicts of interest

There are no conflicts of interest to declare.

## Supplementary Material
